# Comparison between Proteome and Transcriptome Response in Potato (*Solanum tuberosum* L.) Leaves Following Potato Virus Y (PVY) Infection

**DOI:** 10.3390/proteomes5030014

**Published:** 2017-07-06

**Authors:** Tjaša Stare, Katja Stare, Wolfram Weckwerth, Stefanie Wienkoop, Kristina Gruden

**Affiliations:** 1 Department of Biotechnology and Systems Biology, National Institute of Biology, 1000 Ljubljana, Slovenia; katja.stare@nib.si (S.K.); kristina.gruden@nib.si (G.K.); 2 Department of Ecogenomics and Systems Biology, Faculty of Life Sciences, University of Vienna, 1010 Wien, Austria; wolfram.weckwerth@univie.ac.at (W.W.); stefanie.wienkoop@univie.ac.at (W.S.)

**Keywords:** potato virus Y (PVY), proteomics, transcriptomics, potato (*Solanum tuberosum* L.)

## Abstract

Plant diseases caused by viral infection are affecting all major crops. Being an obligate intracellular organisms, chemical control of these pathogens is so far not applied in the field except to control the insect vectors of the viruses. Understanding of molecular responses of plant immunity is therefore economically important, guiding the enforcement of crop resistance. To disentangle complex regulatory mechanisms of the plant immune responses, understanding system as a whole is a must. However, integrating data from different molecular analysis (transcriptomics, proteomics, metabolomics, smallRNA regulation etc.) is not straightforward. We evaluated the response of potato (*Solanum tuberosum* L.) following the infection with potato virus Y (PVY). The response has been analyzed on two molecular levels, with microarray transcriptome analysis and mass spectroscopy-based proteomics. Within this report, we performed detailed analysis of the results on both levels and compared two different approaches for analysis of proteomic data (spectral count versus MaxQuant). To link the data on different molecular levels, each protein was mapped to the corresponding potato transcript according to StNIB paralogue grouping. Only 33% of the proteins mapped to microarray probes in a one-to-one relation and additionally many showed discordance in detected levels of proteins with corresponding transcripts. We discussed functional importance of true biological differences between both levels and showed that the reason for the discordance between transcript and protein abundance lies partly in complexity and structure of biological regulation of proteome and transcriptome and partly in technical issues contributing to it.

## 1. Introduction

Plants are constantly being threatened by a vast range of pests and pathogens, including fungi, bacteria, viruses, nematodes and herbivorous insects [1]. Each stressor elicits a complex cellular and molecular response implemented by the plant in order to prevent damage and ensure survival, often at the expense of growth and yield [2]. An average of 26% of the worldwide crop production is lost each year due to pre-harvest pests and pathogens [1] and the yield losses that can be ascribed to plant viruses are estimated to cost worldwide more than $30 billion annually [3]. The most effective and reliable method of virus management is the enhancement of host resistance [4,5]. To do this efficiently, understanding of molecular mechanisms underlying plant immunity is required. 

Plant immunity is regulated by different phythormones with salicylic acid (SA) being one of the crucial hormones regulating plant defense against viruses [6,7,8]. The response involves complex, fine-tuned reprograming of cell metabolism and involves changes at many different molecular levels reflected finally at the cellular and physiological level [9,10,11]. Systems analyses aim at the integration of multilevel molecular data, such as metabolites, proteins, transcripts and genomic data and subsequent comprehensive multivariate statistical data analysis and mathematical modelling to reveal genome-wide associations and molecular phenotypes [12]. Integration of different levels provides an understanding system as a whole and can disentangle complex regulatory mechanisms of the plant immune responses. 

Virus-induced response of plants has been well studied on the level of transcriptional reprograming (using microarrays or more recently RNA-seq methodologies) [11,13,14,15,16,17], leaving other molecular levels, including proteomics, as a bottle neck [18]. Several approaches can be used to qualitatively and quantitatively study proteome in plants [18]. In a typical proteomic study, the first important step is the protein extraction. This is particularly challenging in plants since they normally contain high concentrations of polysaccharides and polyphenols, which may strongly interfere with the subsequent protein separation and identification steps. Extracted proteins are then separated using either gel-based (2-DE, 2D-DIGE) or chromatography-based methodologies followed by protein identification by mass spectroscopy (MS) or more frequently tandem MS analysis [18]. Among the later ones, spectral counting is a strategy to quantitate proteins in pre-digested protein mixtures analyzed by liquid chromatography online with mass spectrometry [19]. Also, label-free analysis based on precursor peak (MS1) intensities, such as MaxQuant, have become widely used [20]. 

In this report, we compared the response in potato (*Solanum tuberosum* L.) leaves following infection with potato virus Y (PVY) on both transcriptome and proteome levels. Cultivated potato is the most widely grown tuber crop in the world, and the fourth largest food crop in terms of fresh produce, after rice, wheat and tomato [21]. It is susceptible to a wide range of pathogens, among which potato virus Y (PVY) is the most important viral pathogen [22,23]. PVY^NTN^ used in this study is an aggressive isolate responsible for major economic losses of potato [17]. Previously [11], we analyzed the dynamic response of primary metabolism in potato following PVY infection on multiple levels (transcriptional regulation, protein abundance, measuring photosynthetic activity and phenotype) and we showed that SA is an important regulator of the potato defense against PVY. In this scientific correspondence, we will compare two different approaches for analysis of proteomic data (spectral count and MaxQuant). The proteins identified by either of the approaches will be linked to the corresponding genes and connected to Potato Oligo Chip Initiative (POCI) microarray gene expression data. We will address and discuss problems associated to linking data on different molecular levels, arising due to the technical issues as well as due to biology of both type of molecules. We will also discuss functional importance of true biological differences between both levels in regard of detail analysis of targeting nucleotide or amino acid sequences. 

## 2. Methodology

Potato plants of cv. Désirée and its SA-deficient counterpart (NahG-Désirée) [14,24,25] were inoculated with PVY^NTN^ as reported in Stare et al. [11]. Healthy potato plants were grown in stem node tissue culture. Two weeks after node segmentation, they were transferred to soil in a growth chamber and kept at 21 ± 2 °C in the light and 18 ± 2 °C in the dark, at a relative humidity of 75 ± 2% with 70–90 mmol/m^2^/s^2^ radiation (L36W/77 lamp, Osram, Germany) and a 16 h photoperiod. After four weeks of growth in soil, the potato plants were inoculated with PVY^NTN^ (isolate NIB-, GENBANK accession number AJ585342) or mock-inoculated as described in [26]. Three bottom leaves were dusted with carborundum and then inoculated by applying a buffered suspension of sap of either PVY^NTN^-infected (for PVY^NTN^ inoculation) or healthy (for mock-inoculation) potato plants. Samples of inoculated leaves from both groups of plants were collected at 4 dpi, flash frozen in liquid nitrogen, and stored at −80 °C for transcriptome or proteome analysis. Three inoculated leaves of each plant were sampled, one of them being used for transcriptomic analysis and the other two pooled for proteomics [11].

The data has been reanalyzed focusing on linking the changes in protein abundance to changes in mRNA levels and taking the complete dataset into account and not only changes in primary metabolism. Whole transcriptome analysis was performed using custom-designed microarrays (4×44K; AMADID 015425) designed by the Potato Oligo Chip Initiative (POCI) [27] analyzed and statistically evaluated as described [11]. Protein abundance of both genotypes at 4 days following PVY/mock treatment was measured using a one-dimensional nano-flow LC system (UltiMate 3000, Thermo Scientific, Waltham, MA, USA) coupled to an Orbitrap LTQ XL mass spectrometer. 

The proteins were identified using the SEQUEST algorithm and Proteome Discoverer (v 1.3, Thermo Scientific). In-silico peptide lists were generated with the following settings: trypsin as the digestion enzyme and a maximum of three missed cleavages. Mass tolerance was set to 5 ppm for precursor ions and 0.8 Da for fragment ions. Additionally, a decoy database containing reversed sequences was used to estimate the false discovery rate (FDR). Only high confidence (FDR ≤ 0.01%) peptide identifications with a minimum XCorr of 2.0, and proteins with at least two distinct peptides, were considered. The datamatrix of the ProteomeDiscoverer, which contained spectral count information, was used for quantitative analysis. For the analysis purpose, missing values (proteins not identified in the sample) were replaced with 0.5 of the minimum protein expression value [28]. Additionally, proteins were identified and quantified using the label-free quantification (LFQ) data matrix of MaxQuant (v1.5.3.8) software [29]. To search the MS data against a FASTA file, we created from downloads the complete set of available potato sequences, as described [30]. From the results log_2_FC and p-value was calculated and only the differences *p* < 0.05 were considered as significant.

To link proteome and transcriptome data each protein was mapped to the corresponding potato transcript according to StNIB putative paralogue grouping [30] using BLAST algorithms. Sequence analysis for detailed comparison was performed using algorithms implemented in CLC Main Workbench v 6 (QIAGEN, Venlo, The Netherlands). Proteins and transcripts were visualized in *Solanum tuberosum* genome browser [31] and designated to corresponding metabolic pathway or a process according to MapMan ontology [32] adapted for potato [33].

## 3. Results

### 3.1. Proteome Changes in Infected Potato Leaves

**Figure 1:**
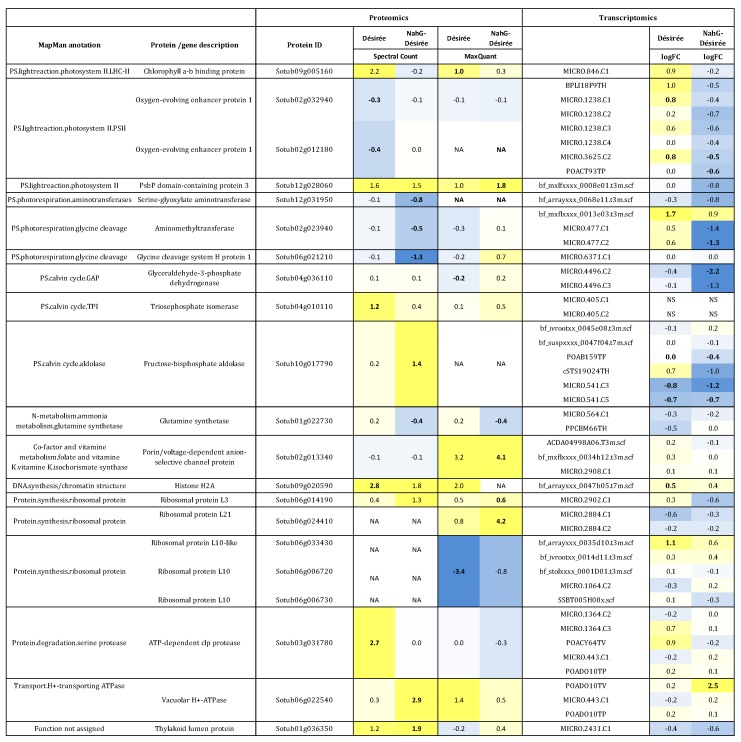
Summary of the differentially abundant proteins in leaves of Désirée and NahG-Désirée plants after infection with PVY and comparison to mRNA expression data. Proteins are grouped according to their function and for each protein the best hit(s) identified according to complete set of available potato sequences are given. Figure shows average ratio (log_2_FC) of the protein abundance or corresponding gene expression (infected versus mock-treated) at 4 dpi. Results for two genotypes are shown (Desiree and NahG-Désirée). As different POCI sequences can represent the same gene, allelic variant or closely related gene family multiple hits were often retrieved per one protein in this database. Proteins are thus linked to multiple results of microarray analysis. Results are color-coded: yellow-up regulation, blue down regulation. NS-not significant, NA-not available (proteins that were not identified). Statistically significant differences (FDR corrected *p* < 0.05) are in bold. PVY = potato virus Y; POCI = Potato Oligo Chip Initiative; FDR = false discovery rate.

Proteome changes of PVY or mock-treated potato plants were previously evaluated in the context of primary metabolism-related changes using spectral count analysis [11]. To evaluate the PVY-induced changes in the context of a whole cell metabolism and processes reprogramming, we have within this report assigned the 339 previously identified and quantified proteins to corresponding metabolic pathway or a process (Figure 1). We found out that the identified proteins are involved in photosynthesis (light reactions, photorespiration, Calvin cycle) and glycolysis as well as protein manipulation (synthesis, degradation, folding), RNA regulation, DNA synthesis and regulation of redox potential. To study the effect of SA, a crucial hormone in plant defence, profiles of two cv. Désirée and its SA-deficient counterpart were compared. Extensive SA-dependent differences were identified and their biological relevance in the context of primary metabolism has been discussed previously [11]. Within this study, we have additionally performed the analysis of proteomics dataset using MaxQuant analytics. With this approach, we identified and quantified 250 proteins out of which 187 were found to be unambiguously identifiable based on proteotypic peptides only. 

Using either of the approaches (MaxQuant or spectral count), 21 proteins among those identified showed significantly altered changes in their abundance following viral infection (Figure 1). PVY infection altered the levels of proteins involved in primary photosynthesis, N-metabolism, DNA synthesis, co-factor and vitamin metabolism, protein synthesis (ribosomal proteins) and protein degradation (protease) and transport (Figure 1). Comparison between different approaches reveals that approximately half of the identified proteins report similar changes of profiles in protein abundance, such being for example chlorophyll a-b binding protein (Sotub09g005160), oxygen-evolving enhancer protein 1 (Sotub02g032940), PsbP domain-containing protein 3 (Sotub12g028060), ribosomal protein L3 (Sotub06g014190) or glutamine synthetase (Sotub01g022730). Not all of them, however, were based on proteotypic peptides only. For instance, quantitative data of Sotub09g005160 are not fully based on proteotypic peptides and peptide IDs are not fully shared between the two search engines. Thus, in the case of porin/voltage-dependent anion-selective channel protein (Sotub02g013340) reported profile of protein abundance differs between both methods. Additionally, some proteins were identified only by one approach (ex. oxygen-evolving enhancer protein 1 (Sotub02g012180) or fructose-bisphosphate aldolase (Sotub10g017790)). 

### 3.2. Integration of Proteome Changes and Reprogramming of Transcriptome

A whole transcriptome analysis was performed on the same plants as sampled for proteomics analysis. PVY infection induced massive reprograming of potato transcriptome [11].

To integrate proteomic data with transcriptomics results, transcripts were linked to each corresponding significantly differentially abundant protein according to either analysis and are shown in Figure 1. Average relative (PVY versus mock) protein abundances (log_2_FC) were compared to corresponding relative changes of concentration of messenger RNA (mRNA). Often there is no straightforward correlation between proteomics and transcriptomics results. To understand these discrepancies better we first analyzed both datasets more in details.

One needs to note that, one-to-one link between protein and transcripts was in our dataset established for only 33% of identified proteins. Often one protein ID is linked to many POCI ID (Figure 1). For example, oxygen-evolving enhancer protein 1 (Sotub02g032940 and Sotub02g012180), aminomethyltransferase (Sotub02g023940), glyceraldehyde-3-phosphate dehydrogenase (Sotub04g036110), fructose biphosphate aldolase (Sotub10g017790), glutamine synthetase (Sotub01g022730), porin/voltage-dependent anion-selective channel (Sotub02g013340), ATP-dependent chloroplast protease (Sotub03g031780.1.1), ribosomal proteins L10 (Sotub06g006720, Sotub06g033430, Sotub06g006730) and vacuolar H+-ATPase (Sotub12g023750) are all linked to multiple transcripts (Figure 1). Some of the transcripts linked to a particular protein can have similar expression profiles while some can be substantially different. 

In the case of chlorophyll a-b binding protein, PsbP domain-containing protein, serine-glyoxylate aminotransferase, glycine cleavage system II protein 1, histone H2A, 50S ribosomal protein L3, and thylakoid lumen protein only one POCI ID was ascribed to each protein; MICRO.846.C1, bf_mxlfxxxx_0008e01.t3m.scf, bf_arrayxxx_0068e11.t3m.scf, MICRO.6371.C1, bf_arrayxxx_0047b05.t7m.scf, MICRO.2902.C1 and MICRO.2431.C1 respectively. Even so, the changes observed on both levels are not necessarily proportional or even showing the same tendency. Gene expression levels of chlorophyll a-b binding protein, serine-glyoxylate aminotransferase and histone H2A followed the tendency of the detected changes on the protein level. For example, up-regulation of histone H2A mRNA or chlorophyll a-b binding protein mRNA in Désirée plants 4 dpi resulted in even stronger increase on protein level (Figure 1). In the case of PsbP domain-containing protein 3 mRNA, however, repression in NahG genotype was linked to strong increase of protein abundance. 

**Figure 2:**
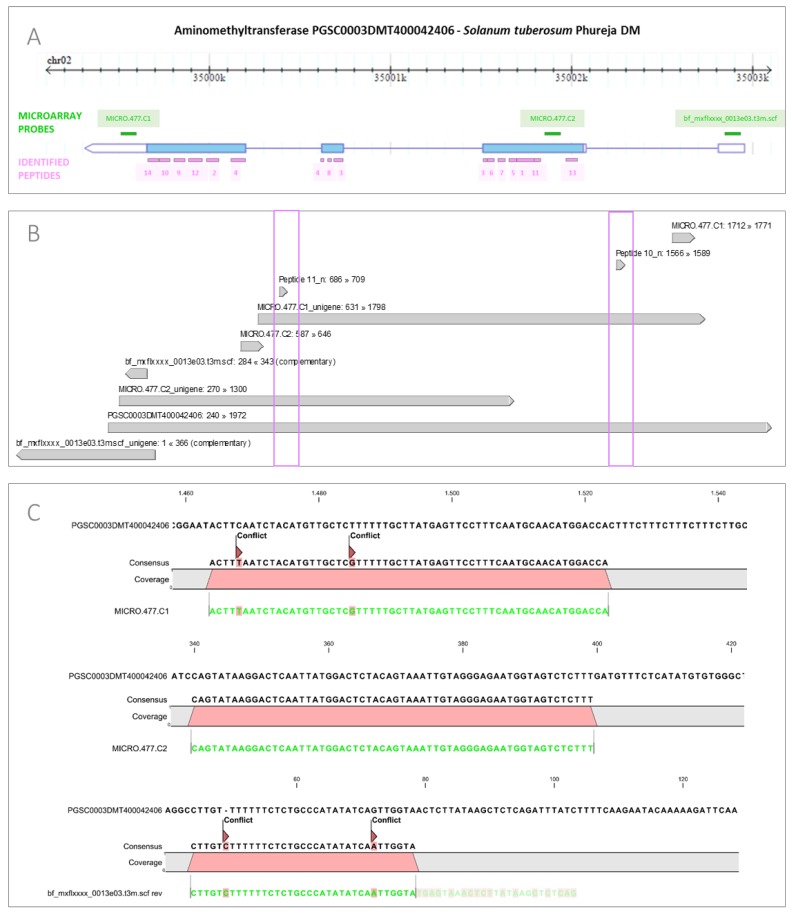
Aminomethyltransferase mapped on MS identified peptides and microarray probes targeting its transcript. (**A**) Identified aminomethyltransferase gene (Sotub02g023940) has been detected in the genome of *Solanum tuberosum Phureja* DM (PGSC0003DMT400042406) at the chromosome 2. MS (spectral count) identified peptides (14) are mapped to the protein (pink). Position of three POCI probes (MICRO.477.C1. MICRO.477.C2, bf_mxflxxxx_0013e03.t3m.scf) hybridizing with the corresponding transcript are shown in green. Light blue is the position of predicted coding sequence of the transcript, blue line denotes intron position, while no fill denotes position of UTRs; (**B**) Alignment of probes and UniGenes corresponding to PGSC0003DMT400042406 transcript. Peptide 10 and 11 are mapped to the contig showing that Peptide 10 is specific to MICRO.477.C1 UniGene, while the other is targeting both UniGenes (MICRO.477.C1 and MICRO.477.C2). Pink squares denote the position of Peptides 10 and 11; (**C**) Visualization of aminomethyltransferase (AMT) transcript with specificity of corresponding microarray probes. Sequences of POCI probes (bf_mxflxxxx_0013e03.t3m.scf, MICRO.477.C1. MICRO.477.C2) are shown in green. A zoom to MICRO.477.C1 (top) sequence shows 2 nt mismatches hybridizing to PGSC0003DMT400042406 transcript, MICRO.477.C2 (middle) probe shows 100%, while probe bf_mxflxxxx_0013e03.t3m.scf (bottom) with 19 SNPs out of the sequence of 60 is not hybridizing to PGSC0003DMT400042406 coding sequence (CDS).

For example, in the case of aminomethyltransferase (Sotub02g023940) 14 peptides matching the sequence were identified by MS analysis (Figure 2A, Supplemental Table S1). They are originating from different positions of the functional protein, spanning from N-terminus to C-terminus of the protein. Two peptides are derived from the junction region between two exons (Peptide 3 and Peptide 4). While 14 identified peptides are covering most of the aminomethyltransferase’s amino acid sequence, not all are mapping to all detected transcripts (from the genome model prediction and from UniGenes). For example, while Peptide 11 is matching to sequence of three transcripts (MICRO.477.C1, MICRO.477.C2, PGSC0003DMT400042406) the Peptide 10 sequence was confirmed only in MICRO.477.C1 and PGSC0003DMT400042406 transcript (Figure 2B). Additional data complexity arises also from specificity of microarray probes. In our microarrays, three probes are targeting corresponding aminomethyltransferase’s mRNAs. Two of them were designed to bind to untranslated region (UTR) of the transcript. One probe is targeting the 5’ UTR (bf_mxflxxxx_0013e03.t3m.scf) and the other one (MICRO.477.C1) 3’ UTR, while MICRO.477.C2 probe is hybridizing to the coding sequence of mRNA. A detailed sequence analysis revealed that MICRO.477.C1 probe is not perfectly matching PGSC0003DMT400042406 transcript; 2 nt mismatches were detected (Figure 2C), while MICRO.477.C2 perfectly aligns to all transcripts in the group, affecting hybridization specificity of the probes. Consequently, MICRO.477.C1 and MICRO.477.C2 probes show very similar but not exactly the same signal profile (Figure 1). On the other hand, mRNA expression profile detected by bf_mxflxxxx_0013e03.t3m.scf probe does not resemble to the other two (MICRO.477.C1, MICRO.477.C2). Detailed analysis shows (Figure 2C) that bf_mxflxxxx_0013e03.t3m.scf probe is not hybridizing to the predicted PGSC0003DMT400042406 transcript sequence as it harbors 19 SNPs out of the sequence of 60 nt long probe and is also in reverse orientation. 

Another example are two proteins annotated as oxygen-evolving enhancer protein 1 (Sotub02g032940, Sotub02g012180, Figure 1). The proteins are showing 92% identity on amino acid level and according to their description perform similar function (Supplemental Figure S1A). Genes encoding for both proteins were identified as paralogues [34] residing at different locations in the potato genome. For both paralogues, protein abundances are similarly affected by PVY infection. Both proteins, however, link to the same set of microarray probes (BPLI18F9TH, MICRO.1238.C1, MICRO.1238.C2, MICRO.1238.C3, MICRO.1238.C4, MICRO.3625.C2, POACT93TP; Figure 1). Each of the transcripts has its unique expression profile. More detailed analysis of probe sequences shows that they do not hybridize efficiently to neither of the protein’s predicted mRNA (neither Sotub02g032940 nor Sotub02g012180 transcripts, Supplemental Figure S1B), resulting in different expression patterns between mRNA and protein level. The data obtained on protein level can thus not be matched to the transcriptome data as the microarray probes were not designed to detect transcripts coding for those two particular proteins (Supplemental Figure S1).

## 4. Discussion

Analyzing and comparing potato response to PVY infection on two molecular levels revealed that translation between transcriptomics and proteomics data is not trivial. Changes on the level of transcriptome are much stronger than those on the level of proteome [11]. While severe reprogramming of potato transcriptome was detected, changes of protein abundance are not so pronounced. Proteome is generally more stable, and only some of the transcriptional perturbations are reflected at the level of the proteins [35,36]. Comparison of sensitivity of both applied methods showed that expression values of 40,000 probes, representing 18,836 genes, were above the limit of quantification, whereas this was true for only 339 proteins [11]. Therefore, it is possible that due to the limited protein identification sensitivity, changes in abundance of some proteins were missed. Most of the detected proteins and the ones that significantly change the abundance correspond to photosynthesis-related processes such as light reaction and photorespiration (Figure 1). Correspondingly, also other studies of plant–virus interaction commonly report changes in proteins involved in photosynthesis metabolism [18]. Shot-gun proteomics technique favors the detection of abundant proteins over the detection of proteins that are found in minor concentrations, since the peptides derived from the abundant proteins have higher probability to be sampled by the MS instrument [37]. Ribulose-1,5-bisphosphate carboxylase oxygenase (Rubisco) is the prevalent protein in plant leaves. As a major enzyme involved in carbon fixation, Rubisco consists of 30 to 50% of total plant protein in green tissues and greatly impacts sensitivity of other protein identification as well as their quantification [38,39]. Rubisco depletion methods [38,40,41] are the pretreatments to be considered to eliminate this problem. However, all of the depletion procedures are sample manipulation that result in bias in extraction, which is a really major issue for establishing confident comparisons between samples and significant biological conclusions [42].

In addition to the spectral count approach, label-free analysis based on precursor peak (MS1) intensities have become very popular [20] approaches for MS-based quantitative proteomic analysis. The most frequently used software that implements MS1 quantification is MaxQuant [29] which detects features by fitting a Gaussian peak shape to three dimensions and then estimates peptide intensity as the volume of this 3D feature [43]. Additional analysis of proteomic dataset was performed using MaxQuant software, to avoid potential bias from single-approach analysis. Significant changes in abundance of proteins involved in photosynthesis have been detected with both approaches (Figure 1). Similarly to spectral count approach, also with MaxQuant analysis the discrepancies between protein abundance and corresponding gene expression level occurs (Figure 1). 

Protein identification using spectral counting or MaxQuant approach depends on the completeness of protein sequencing databases [44]. Therefore, lack of quality genome sequence also interferes with proteomics analysis. While genomes of many model organisms including some plants are available for most studied genotypes [45], this is not the case for potato. With release of *Solanum tuberosum* genome in 2011 [31], the potato community has gained an important high-quality draft potato genome sequence. The genome sequence is derived from homozygous diploid genotype of potato, differing from commercially grown potato cultivars which are highly heterozygous autotetraploids (2n = 4x = 48) belonging to different subspecies (*Solanum tuberosum phureja* versus *Solanum tuberosum tuberosum*). Taking into account also cultivar-specific genome variations, the reference sequences can vary greatly from the ones in the particular experimental material. In such cases, customized protein libraries based on translation of genomes supplemented with RNAseq can be a useful alternative [44]. While we have indeed created a complete set of available potato sequences [30], to help minimize this issue, the complete reference transcriptome sequence for cv. Désirée is, however, still not available. 

Incomplete genome sequence can introduce bias also in transcriptomic analysis. As POCI sequences can represent different allelic variant of the same gene or closely related gene family member, multiple hits were often retrieved per one protein resulting in linking one protein to multiple POCI identifiers (Figure 1). Additional discrepancies can occur as a consequence of partial sequence matching both on the level of transcript and protein identification. Specificity of microarray-based detection is acquired through 60 nt long probes, each designed to target one particular potato UniGene. In the case of aminomethyltransferase, two probes (MICRO.477.C1 and MICRO.477.C2) targeting the same transcript were showed to have similar, but not exactly the same, expression profile (Figure 1). One of the probes (MICRO.477.C1) has 2 nt sequence mismatches according to reference genome while the other is a perfect match (Figure 2). Sequence mismatches will affect the specificity of hybridization to probes spotted on microarrays and therefore the accuracy with which microarray-based assays report the gene expression levels. Signal intensity in probes containing mismatches can be reduced or even diminished. The extent of these effects is difficult to predict, as the position of the mismatch in the probe sequence in combination with a given sequence contributes to efficiency of hybridization. Mismatches near the middle of probes are associated with a greater reduction in signal intensity than those near the end of probes [46]. On the other hand MS spectral identification is confirmed when two detected peptides exactly match the protein sequence. However, the detected peptides and spotted probes often do not align to the same sequence position of the protein or transcript respectively. This was shown in the case of aminomethyltransferase (Figure 2A) where it is clearly seen that nucleotide probes and MS-detected peptides do not target the same position of aminomethyltransferase sequence. Additionally, only two probes hybridize to the aminomethyltransferase CDS, while probe bf_mxflxxxx_0013e03.t3m.scf is incorrectly oriented including several mismatches (Figure 2B,C). It has been previously reported that plant microarrays that do not have fully annotated genomes typically include multiple probes that have incorrect orientation and, consequently, do not produce meaningful data [47]. 

Additionally, highly homologues proteins can introduce biases. Especially potato seems to have rather large numbers of gene families [30]. As in spectral counting proteomics, we require only two peptides per protein to identify the protein, we are basically working at the level of paralogues identification.

The biological variability between analyzed samples is another important aspect to consider when comparing proteome and transcriptome response. In our experimental setup, simultaneous sampling for both analyses was performed in order to avoid biases resulting from time and batch effects and minimize biological variability. However, to ensure enough leaf material for different analysis, we sampled several neighboring leaves of the same plant. Potential effect of leaf-to-leaf variations can be an additional reason for discordance among results. Even though optimization procedures would need to be applied to insure enough biological material, methods for combined isolation of proteins and RNA molecules such as that described by Valledor and coworkers [12] could help to eliminate this bias. 

Additionally, the discrepancies between transcript and protein level can also be the consequence of specific biological process. Protein abundances reflect a dynamic balance among production and maintenance of cellular protein, reflecting series of linked processes; transcription, processing and degradation of mRNAs to the translation, localization, modification and programmed destruction of the proteins [48]. One of the biological reasons for low correlation between expression profiles of transcriptome and proteome data lies in posttranscriptional regulation [48] mechanisms. Gene regulation orchestrated by micro RNAs (miRNAs) is one of the posttranscriptional regulation mechanisms. These short non-coding RNA species, 21–24 nt in length, regulate mRNA via two mechanisms; translational inhibition and mRNA degradation. If the translation inhibition occurs, the mRNA would still be detected even though it would not be translated to protein [44,49]. Quantitative studies of circadian rhythm revealed another important posttranscriptional mechanism regulating efficiency of translation via activity of ribosomal proteins and translation initiation factors. PVY infection of potato plants induced changes in abundance of ribosomal proteins (Figure 1). These proteins help stabilize the formation of the functional ribosome around the start codon and additionally provide regulatory mechanisms in translation efficiency. Regulation via phosphorylation of elongation factors or their binding partners controls initiation phase of protein synthesis, thereby controlling protein synthesis independent of mRNA expression [50,51,52]. In mammals kinase-mediated phosphorylation of elongation factor is elicited upon viral infection and other stressors (ER stress, heme deficiency, or amino acid deprivation) [53] and ER stress was detected also in Potyviridae infected plants [54]. The third molecular mechanism leading to apparent discrepancy is explained by differences in protein turnover rates [34,55]. In mammalian cells, mRNAs are produced at a much lower rate than proteins are; on average, a cell produces two copies of a given mRNA per hour, whereas it produces dozens of copies of the corresponding protein per mRNA in the same time frame. Similarly, mRNAs are less stable than proteins, with an average half-life of 2.6–7 h versus 46 h for proteins [48].

## 5. Conclusions

Evaluating cell behavior on different molecular levels is crucial for understanding system as a whole. Transcriptome and proteome analysis are two important complementary molecular aspects to be included in such comprehensive studies. Integration of both datasets helps to understand the functional relevance of changes in different processes. We compared the relation between these two levels in the case of potato response to PVY. Although there is sometimes an encouragingly simple relationship between changes of transcripts and proteins and changes in downstream biological functions, quite often this is not the case. This apparent disconnect lies partly in the complexity of regulation of biological processes and partly in technical issues. In summary, one should keep in mind that the limitations discussed herein when interpreting the results and often the requirement of detailed inspection of the results in case-by-case manner is needed.

## Supplementary Materials

The following are available online at http://www.mdpi.com/2227-7382/5/3/14, Figure S1: Alignment of oxygen-evolving enhancer protein 1 (Sotub02g032940, Sotub02g012180) sequences, Table S1: Identified peptides and probes as targets of aminomethyltransferase.
