# Supplementary material for: Comparison between Proteome and Transcriptome Response in Potato (Solanum tuberosum L.) Leaves Following Potato Virus Y (PVY) Infection
Source: Proteomes. 2017 Jul 6;5(3):14. doi: 10.3390/proteomes5030014 (PMC5620531; doi:10.3390/proteomes5030014)

Table S1:

Peptide ID	Amino acid sequence	Probe ID	Nucleotide sequence
**Peptide 1**	LVIADVAGLAPGTGSLTVFTNEK	**bf_mxflxxxx_0013e03.t3m.scf**	CTGAGAGCTTATAAGAGTTTACTCATACCAATTGATATATGGGCAGAGAAAAAAGACAAG
**Peptide 2**	AEGGFLGAEVILK		
**Peptide 3**	SLLALQGPLAAPVLQYLTK		
**Peptide 4**	TGYTGEDGFEISVPSENALDLAK		
**Peptide 5**	GGAIDDSVVTK	**MICRO.477.C1**	ACTTTAATCTACATGTTGCTCGTTTTTGCTTATGAGTTCCTTTCAATGCAACATGGACCA
**Peptide 6**	GGDVSWHIHDER		
**Peptide 7**	DLAHIEEHMK		
**Peptide 8**	MYFGEFR		
**Peptide 9**	NIAMGYVK		
**Peptide 10**	SYDGVVTK		
**Peptide 11**	DTIPFLEK	**MICRO.477.C2**	CAGTATAAGGACTCAATTATGGACTCTACAGTAAATTGTAGGGAGAATGGTAGTCTCTTT
**Peptide 12**	VGFFSSGPPPR		
**Peptide 13**	TVLYDFHVVNGGK		
**Peptide 14**	MPFVPTK		

Figure S1

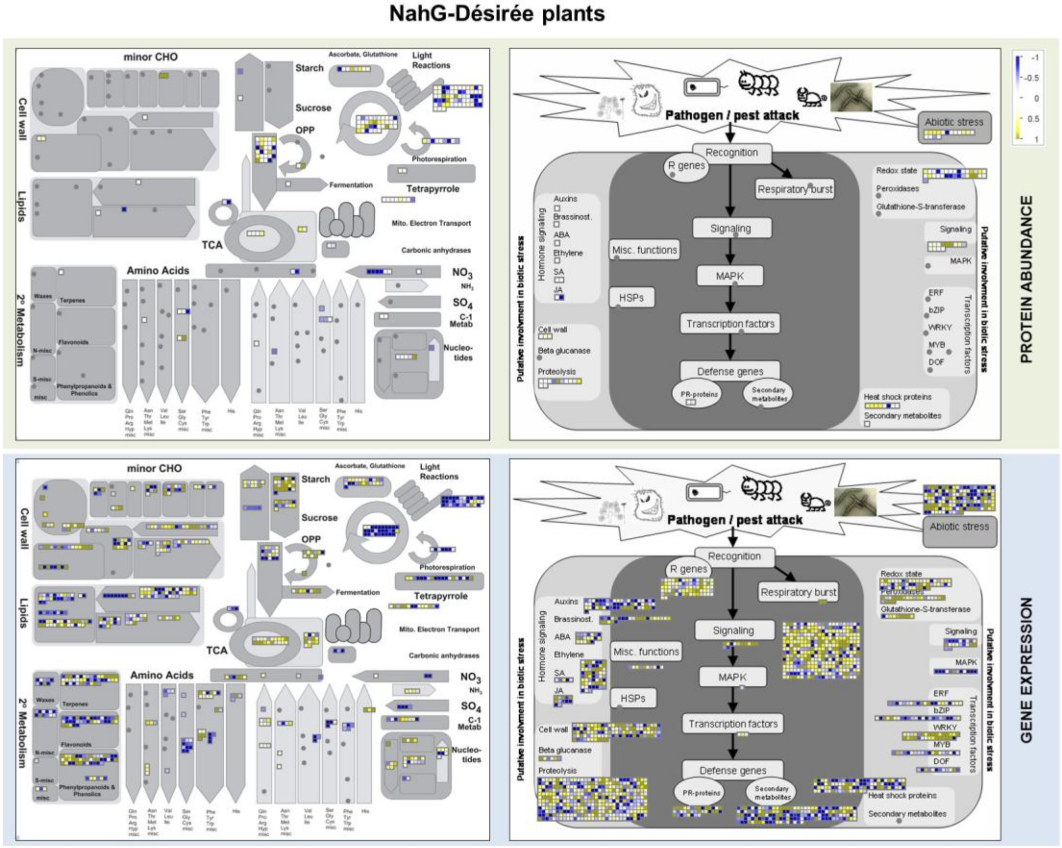

Supplement: Supplementary file 1 [file proteomes-05-00014-s001.pdf]
